# Real-world outcomes of lower lenvatinib doses in advanced neuroendocrine tumors: a multinational retrospective study

**DOI:** 10.1530/EO-25-0076

**Published:** 2025-12-03

**Authors:** G R Almeida, G K Zanetta, J M O’Connor, M J Barros, T C Felismino, R F Weschenfelder, A Bel-Ange, S Grozinsky-Glasberg, P R Riechelmann

**Affiliations:** ^1^ AC Camargo Cancer Center, São Paulo, Brazil; ^2^ Instituto Alexander Fleming, Buenos Aires, Argentina; ^3^ Hospital Moinhos de Vento, Porto Alegre, Brazil; ^4^ NET Unit, ENETS Center of Excellence, Division of Internal Medicine, Hadassah Medical Center and Faculty of Medicine, The Hebrew University, Jerusalem, Israel

**Keywords:** neuroendocrine tumors, metastasis, carcinoids

## Abstract

**Objective:**

Neuroendocrine tumors (NET) are heterogeneous neoplasms with increasing incidence and limited treatment options for advanced, progressive disease. Lenvatinib, a multitargeted TKI, demonstrated high efficacy but substantial toxicity at the standard 24 mg/day in the phase II TALENT trial. This study evaluates real-world efficacy and safety of lower-dose lenvatinib in patients with grade 1–3 GEP or thoracic NET.

**Design:**

Retrospective multinational cohort study.

**Methods:**

Twenty-two patients from Brazil, Argentina, and Israel with grade 1–3 NET and radiologic progression received lenvatinib (starting doses per physician discretion) between March 2021 and September 2024. The primary endpoint was progression-free survival (PFS); secondary endpoints were overall survival (OS) and grade ≥3 adverse events (AEs). Exploratory endpoints included objective response rate (ORR) and disease control rate (DCR). Kaplan–Meier methods estimated time-to-event outcomes; AEs were graded per CTCAE v5.0.

**Results:**

Median age was 60 years (range: 36–81); primary sites were pancreatic (27%), gastrointestinal (54.5%), and thoracic (18.2%). Grades: G1 (23%), G2 (45%), G3 (31.8%). Starting doses ranged 8–24 mg/day, most often 10 mg (32%) or 8 mg (27%); 45% required reductions. Mean daily dose was 11.8 mg (±4.7). After 16.8 months median follow-up, mPFS was 13 months (IQR: 8.8–17.9) and mOS 16 months (IQR: 12.6–18.5). Among 22 evaluable patients, ORR was 31.8% and DCR 90.9%. Grade 3–4 AEs occurred in 22%; most were grade 1–2 (fatigue 31.8%, hypertension 22.7%).

**Conclusions:**

Lenvatinib (average of 10–12 mg/day) showed meaningful antitumor activity and improved tolerability in advanced GEP and thoracic NET, supporting individualized dosing to enhance safety without compromising efficacy.

## Introduction

Neuroendocrine tumors (NET) are rare neoplasms arising from neuroendocrine cells, most frequently occurring in the gastroenteropancreatic (GEP) region. Although traditionally considered uncommon, the incidence of NET has been steadily rising over the past decades, partly due to increased awareness and improved diagnostic techniques (1, 2, 3, 4). These tumors are heterogeneous, with varying grades (G1–G3) and clinical behaviors, often presenting challenges in managing advanced, progressive disease. Systemic therapies, including somatostatin analogs (SSAs), peptide receptor radionuclide therapy (PRRT), chemotherapy, and tyrosine kinase inhibitors (TKIs), are employed to control tumor growth and manage symptoms (5). However, resistance to these therapies remains a significant barrier, particularly because patients with advanced GEP NET live for several years, necessitating novel treatment strategies to achieve tumor control while minimizing toxicity (6, 7).

Lenvatinib, a TKI that targets VEGFR1–3, FGFR1–4, PDGFR alpha, among other kinases, has emerged as a promising therapy for advanced GEP-NET (8, 9, 10). The phase II TALENT trial evaluated lenvatinib at 24 mg daily in patients with advanced, progressive grade 1–2 GEP-NET who had documented radiological progression after treatment with targeted agents (pancreatic NET) or SSA (gastrointestinal NET). The trial reported a centrally confirmed objective response rate (ORR) of 29.9%, marking the highest ORR observed with a TKI in this setting (7, 11, 12), and a median progression-free survival (PFS) of 15.7 months. These findings highlighted lenvatinib’s potential as an effective therapy in pretreated patients, potentially overcoming resistance to prior anti-VEGF therapies (7, 8).

Despite its efficacy, lenvatinib at the tested dose of 24 mg PO daily was associated with significant toxicity in the TALENT trial, with 93.7% of patients requiring dose reductions or interruptions due to adverse events such as fatigue, hypertension, and diarrhea. This high rate of toxicity has prompted clinicians to explore lower starting doses or dose adjustments in clinical practice to improve tolerability while maintaining antitumor activity. However, the safety and effectiveness of lower doses of lenvatinib remain uncertain. In addition, the antitumor effects of lenvatinib have not been explored in patients with advanced thoracic NET or grade 3 NET. This retrospective multinational cohort study aimed to evaluate the real-world efficacy and toxicity of lower doses of lenvatinib in patients with advanced, progressive GEP or thoracic NET, hypothesizing that lower doses can provide comparable tumor control, improved tolerability, and potential cost savings compared to the standard PO 24 mg daily dose.

## Methods

This retrospective, multinational cohort study included patients from institutions across Brazil, Argentina, and Israel. Eligible patients had histologically confirmed grade 1–3 GEP or thoracic NET, according to the WHO classification of 2022 (13), with evidence of radiological disease progression and who received at least one dose of lenvatinib. Treatment with lenvatinib was administered at the discretion of the treating physician, with starting doses and subsequent adjustments based on individual patient tolerability and clinical judgment.

The primary endpoint was PFS, defined as the interval from the initiation of lenvatinib to the date of radiological disease progression, as per RECIST 1.1, or death from any cause, whichever occurred first. Patients who were alive and progression-free at the time of data analysis were censored at their last follow-up. Secondary endpoints included overall survival (OS), calculated from the start of lenvatinib to death from any cause, with censoring at the last known alive date for surviving patients; and the incidence of grade 3 or higher adverse events, assessed using the Common Terminology Criteria for Adverse Events (CTCAE) version 5.0. Additional exploratory analyses included ORR and disease control rate (DCR), evaluated through radiological assessments performed as part of routine clinical care.

Clinical and demographic data were extracted from electronic medical records and included age, sex, primary tumor site, tumor grade, prior treatments, lenvatinib dosing details (starting dose, dose reductions, and mean daily dose), and adverse event profiles. The study was conducted in accordance with the Declaration of Helsinki and local regulations. Institutional review board/ethics committee approval was obtained at each participating center, with a waiver of informed consent owing to the retrospective design and use of de-identified data: AC Camargo Cancer Center, Instituto Alexander Fleming, Hospital Moinhos de Vento, and Hadassah Medical Center. Statistical analyses were conducted using descriptive statistics for baseline characteristics and Kaplan–Meier estimates for time-to-event endpoints (PFS and OS), with the log-rank test used to compare between-group differences. Cox proportional hazards models were used to estimate hazard ratios for each variable. Adverse event frequencies were reported as proportions. All statistical analyses, graphs, and tables were performed using RStudio v2024.04.2.

## Results

Our study retrospectively evaluated 22 patients diagnosed with NET between 2007 and 2020 who started lenvatinib between March 2021 and September 2024. The median age at lenvatinib start was 60 years (range: 36–81). The most common primary tumor sites were pancreas (6; 27%) and gastrointestinal (12; 54.5%), followed by thoracic sites (three lung and one thymus; 18.2%). Most patients had either grade 2 (*n* = 10; 45%) or grade 3 (*n* = 7; 31.8%) tumors (Table 1).

**Table 1:** Patient characteristics (*n* = 22).

Characteristics	*n* (%)
Age at lenvatinib, years (median, range)	60.5 (36–81)
**ECOG performance status**	
0–1	7 (100%)
**Sex**	
Female	10 (45.5%)
Male	12 (54.5%)
**Race**	
White	18 (81.8%)
Black	2 (9.1%)
Asian	1 (4.5%)
Other	1 (4.5%)
**Primary tumor origin**	
GI	12 (54.5%)
Pancreas	6 (27.3%)
Thoracic	4 (18.2%)
**Tumor grade at diagnosis**	
G1	2 (9.1%)
G2	13 (59.1%)
G3	7 (31.8%)
**Ki-67 index**	
≤10%	9 (40.9%)
>10%	13 (59.1%)
Metastasis sites*	
Liver	19 (40.4%)
Lymph nodes	13 (27.7%)
Lung	4 (8.5%)
Bone	11 (23.4%)
**Prior tyrosine kinase inhibitor**	
Yes	6 (27.3%)
No	16 (72.7%)
**Lenvatinib dose reduction**	
Yes	10 (45.5%)
No	12 (54.5%)

* Patients could have more than one site of metastasis.

**Figure 1:**
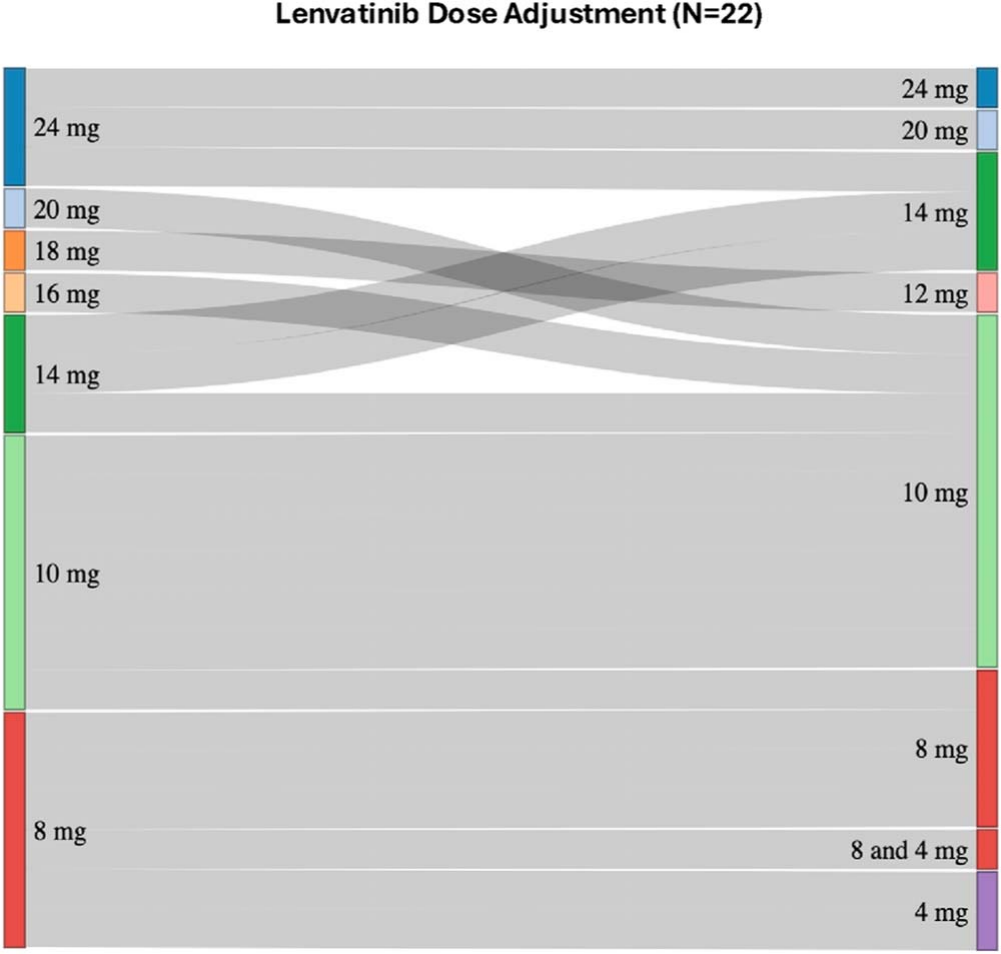
Lenvatinib dose adjustment (*n* = 22). The diagram shows the distribution of patients according to their starting daily dose of lenvatinib (left) and the final or reduced dose (right). The width of each band is proportional to the number of patients undergoing that specific dose adjustment.

**Figure 2:**
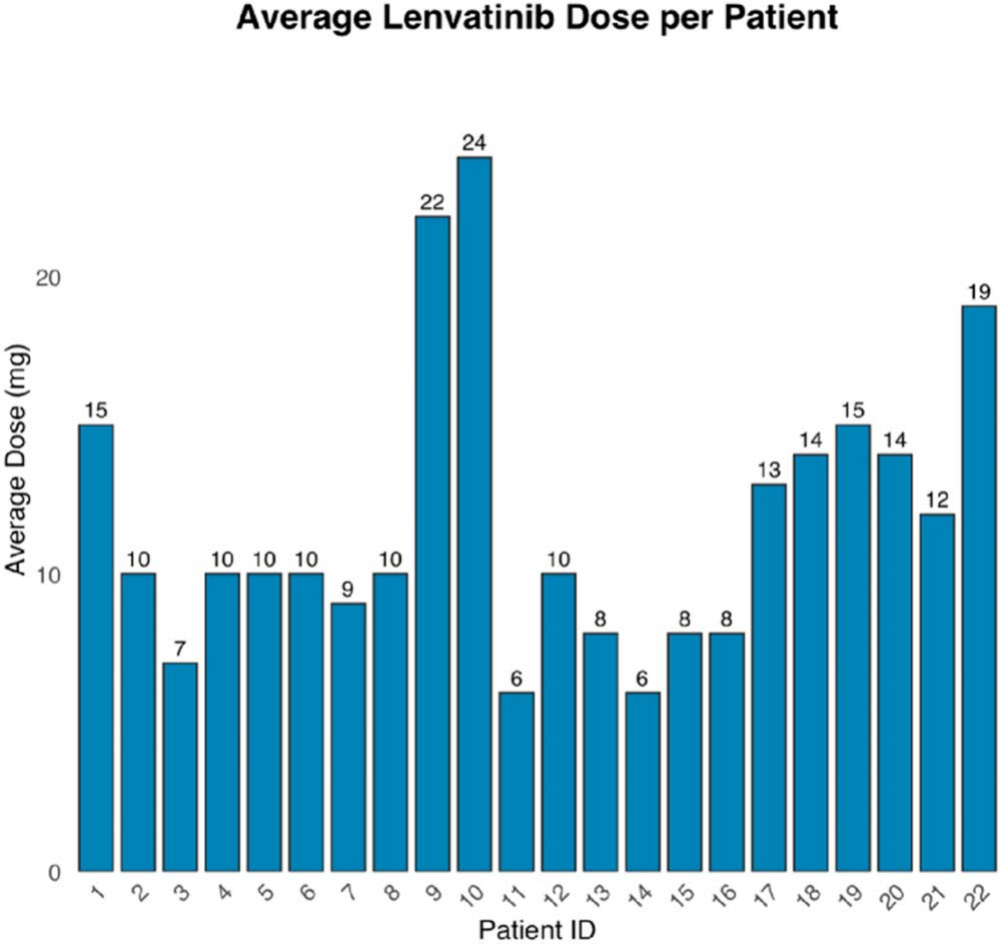
Average lenvatinib dose per patient. Bar plots show the mean daily dose of lenvatinib for each of the 22 patients included in the study. Average doses ranged from 6 to 24 mg per day, with most patients receiving between 8 and 14 mg.

The main reason for upfront dose reductions was the expected toxicity of lenvatinib at higher doses based on physicians’ experience with other types of solid tumors, regardless of patients’ clinical conditions or frailty. The most frequent starting doses of lenvatinib were 10 mg (7; 32%) and 8 mg (6; 27%), followed by 14–18 mg (5; 23%) and 20–24 mg (4; 19%) daily (Fig. 1). Dose reductions were required in ten patients (45%), including four patients who started on 20–24 mg/day, two who initiated 14–18 mg/day, and four at 8–10 mg/day. Reduced doses ranged from 4 to 20 mg/day, with a mean of 9.5 mg/day (Fig. 2). The overall mean daily dose of lenvatinib across all patients was 11.8 mg (standard deviation (SD) ±4.7; range: 4–24 mg/day), with means of 10, 13, and 12.5 mg for patients achieving partial response, upfront progression, or stable disease, respectively. The median time to dose reduction was 2 months after treatment initiation, with most adjustments occurring during this period; only one patient required a reduction beyond 2 months (range: 0–12 months). Patients received a median of four prior lines of systemic therapy (range: 2–7). All patients received SSA, 64% chemotherapy, 41% PRRT, 41% everolimus, and 14% sunitinib. A full listing of prior lines by patient is provided in Table 2 in the Supplementary Appendix (see section on Supplementary materials given at the end of the article).

**Table 2:** Treatment-related toxicities.

Treatment-related toxicities	*n* (%)
**Grade 3 toxicity**	
Yes	4 (18.2%)
No	18 (81.8%)
**Grade 4 toxicity**	
Yes	1 (4.5%)
No	21 (95.5%)
**Fatigue**	
No	10 (45.5%)
G1	7 (31.8%)
G2	5 (22.7%)
**Hypertension**	
No	17 (77.3%)
G1	3 (13.6%)
Yes, not graded	2 (9.1%)
**Other toxicities**	
G1 diarrhea	1 (10%)
G1 HFS	3 (30%)
G1 mucositis	1 (10%)
G2 nausea	1 (10%)
Hepatotoxicity	1 (10%)
Myelotoxicity	3 (30%)

Adverse events were graded according to CTCAE v5.0.

Percentages are based on the total study population (*n* = 22).

**Figure 3:**
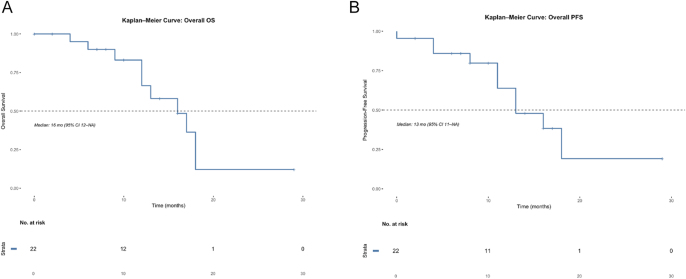
OS and PFS in the entire cohort. Kaplan–Meier estimates of OS (panel A) and PFS (panel B) in all 22 patients treated with lenvatinib. Numbers at risk are shown below each plot.

**Figure 4:**
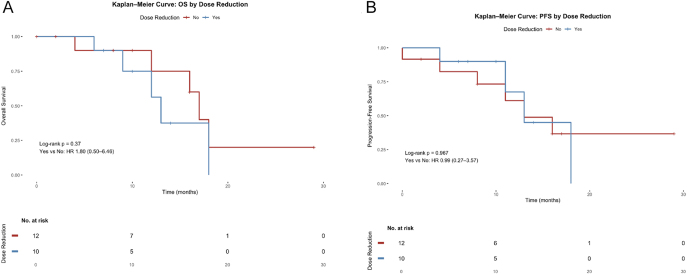
Survival according to lenvatinib dose reduction. Kaplan–Meier curves show OS (panel A) and PFS (panel B) stratified by whether patients required dose reduction. No significant differences were observed between groups.

**Figure 5:**
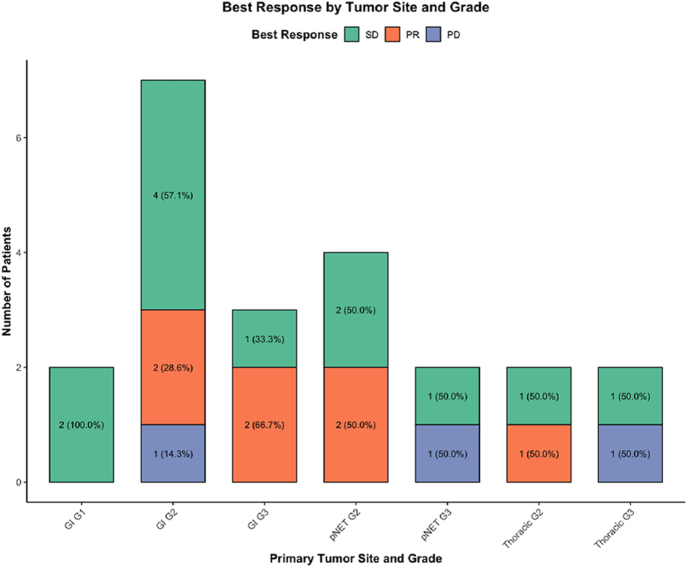
Stacked bar plots show best radiologic response to lenvatinib by primary tumor site and grade. The proportion of patients achieving stable disease (SD, green), partial response (PR, orange), and progressive disease (PD, blue) is displayed for each subgroup. Percentages are indicated within each bar and are based on the total number of patients in the corresponding subgroup.

After a median follow-up of 16.8 months, the median PFS (mPFS) was 13 months (interquartile range (IQR): 8.8–17.9) and median OS (mOS) was 16 months (IQR: 12.6–18.5). Exploratory analysis showed no significant difference in PFS times (95% CI: 0.27 – 3.57) or OS (95% CI: 0.50 – 6.46) between patients who underwent dose reduction and those who did not (Figs 3 and 4). Of the 22 patients evaluated for response, 7 (32%) achieved an objective response, 12 (54%) had stable disease, and 3 (14%) experienced upfront progression, yielding an ORR of 31.8% and DCR of 90.9%. Among the seven patients who achieved an objective response, two had pancreatic NET, four had a small bowel primary, and one patient had lung NET (Fig. 5).

Grade 3 or 4 adverse events occurred in five patients (22%), three of whom started lenvatinib at 20–24 mg/day (Table 2). Among these, grade 3 events included one patient with AST/ALT elevation, two with thrombocytopenia, and one with vomiting; the single grade 4 event was myelotoxicity. The most common adverse events were grade 1–2 fatigue (7; 31%) and hypertension (3; 14%). Fatigue was reported in 12 patients overall, including seven with grade 1 (31.8%) and five with grade 2 (22.7%). Hypertension occurred in five patients (22.7%), three experiencing grade 1 and two with no grade recorded. Other low-grade toxicities included hand-foot syndrome (HFS) in three patients (13.6%), and isolated cases of grade 1 diarrhea (1; 4.5%), grade 1 mucositis (1; 4.5%), grade 2 nausea (1; 4.5%), hepatotoxicity (1; 4.5%), and myelotoxicity (3; 13.6%). No patients permanently discontinued treatment due to toxicity.

## Discussion

Our study suggests that lower doses of lenvatinib can provide clinically meaningful tumor control with reduced toxicity when indirectly compared with standard doses. The mean daily oral dose of nearly 12 mg was associated with PFS and ORRs similar to those reported in the TALENT trial, which tested 24 mg PO, but with less toxicity. Although exploratory, our data also indicate that lenvatinib may be active in patients with grade 3 tumors and potentially in those with advanced lung NET.

The TALENT trial, a multicenter, open-label, single-arm phase II trial of 111 patients with advanced grade 1–2 GEP-NET, reported an ORR of 29.9%, mPFS of 15.7 months, and mOS of 32 months in the pancreatic NET (panNET) group, and not reached in the gastrointestinal NET (GI-NET) group, using lenvatinib at 24 mg/day (8). Notably, the panNET cohort achieved an ORR of 44.2%, while the GI-NET cohort had a lower ORR of 16.4%. Similarly, our study observed an ORR of 31.8% and an mPFS of 13 months across a mixed GEP and thoracic NET population, suggesting that lower doses maintain antitumor activity. A key distinction between our study and the TALENT trial lies in the toxicity profile. In the TALENT trial, dose reductions were required in 81.1% of patients, and 92.8% experienced temporary treatment interruptions. The most prevalent grade 3/4 adverse events included hypertension (22.7%), asthenia (13.6%), and diarrhea (10.9%), with 14.4% of patients discontinuing treatment due to toxicity. In contrast, our study reported a lower rate of dose reductions (45%) and treatment interruptions, with grade 3/4 adverse events observed in 22% of patients. Common adverse events in our study were primarily grade 1–2, including fatigue (31%) and hypertension (14%), and no patient discontinued treatment due to toxicity. The lower incidence of severe toxicity in our cohort is likely due to the use of lower initial lenvatinib doses and individualized dose adjustments, resulting in a mean daily dose of 11.8 mg/day. These findings suggest that lenvatinib at reduced doses can achieve a more favorable balance between efficacy and tolerability.

Other studies of lenvatinib in NET provide additional context. A phase II trial by Al-Toubah *et al.* (2024) investigated lenvatinib combined with pembrolizumab in 20 patients with advanced, well-differentiated NET (excluding panNET), reporting a lower ORR of 10% and a mPFS of 8 months (14). The reduced efficacy in this study may be attributed to the exclusion of panNET, which consistently show higher response rates to TKI (8, 11, 12, 14), including lenvatinib (e.g., 44.2% in TALENT), and the limited activity of immunotherapy in well-differentiated NET. Our study’s inclusion of panNET (27.3% of patients) likely contributed to the higher ORR and PFS, reinforcing the importance of tumor site in treatment response. In addition, we observed response with lenvatinib in thoracic NET, a finding that expands the potential applicability beyond GEP-NET. This is particularly noteworthy given the limited data on lenvatinib efficacy in thoracic NET, highlighting the need for further investigation.

The Canadian real-world series by Vasconcelos *et al.* evaluated lenvatinib in 33 patients with predominantly G1–2 GEP-NET and closely mirrored our findings, with median daily doses of 8–12 mg, mPFS 11.9 months, mOS 17.5 months, ORR 21.9%, and DCR 87.5%, with hypertension (60.6%) and fatigue (39.4%) as the most frequent adverse events (15). In our cohort, treated within a similar dosing range, we observed comparable efficacy (mPFS 13.0 months, mOS 16.0 months, ORR 31.8%, DCR 90.9%) and safety, while also demonstrating meaningful activity in underrepresented subgroups (grade 3 and thoracic NET). Together, these data support the use of reduced-dose lenvatinib in NET to maintain antitumor efficacy with a more favorable toxicity profile.

As further evidence of anti-angiogenic TKI activity in NET, the phase III CABINET trial randomized pre-treated patients with G1–3 extrapancreatic (*n* = 203) and pancreatic (*n* = 95) NET to cabozantinib 60 mg/day versus placebo (11). In extrapancreatic NET, cabozantinib extended mPFS from 3.9 to 8.4 months, and in pancreatic NET from 4.4 to 13.8 months. Grade ≥3 treatment-related adverse events occurred in 62–65%, mainly hypertension, fatigue, and diarrhea. Notably, 24% of patients in the extrapancreatic cohort had thoracic primaries, and 8% of the overall population had G3 NET. While cabozantinib and lenvatinib differ in their kinase selectivity, these findings support the broader principle that anti-angiogenic TKIs can be active across NET subtypes, including thoracic and higher-grade tumors, and underscore the need for regimens with a more favorable toxicity profile.

In the evolving treatment landscape for advanced NET, the optimal sequencing of therapies remains uncertain. On the basis of our data, lenvatinib – particularly at reduced, individualized doses – appears effective in heavily pretreated patients, including those with progression after other targeted therapies, particularly when an objective response is desired or when alternative options are scarce or limited by toxicity. In that setting, we think lenvatinib could be used either before or after cabozantinib. Although lenvatinib demonstrated efficacy in a phase II trial, its use remains off-label and is not yet endorsed by formal guidelines due to limited evidence. Prospective randomized studies are warranted to better define its therapeutic role and optimal sequencing among anti-angiogenic TKIs.

Our study has some limitations. First, the retrospective design and small sample size limit the generalizability of our findings and introduce potential selection bias. The heterogeneity of the patient population, including various primary tumor sites and tumor grades, may also confound the interpretation of efficacy outcomes. In addition, missing or incomplete data in medical records limited the availability of detailed information on some variables, such as outcomes from prior therapies. Finally, the reasons for upfront dose reductions relied on the treating physicians, with substantial variability, precluding recommendations on an optimal dosing strategy. The main reason for upfront dose reductions was the expected toxicity of lenvatinib at higher doses, regardless of patients’ clinical conditions or frailty. Therefore, a selection bias towards worse prognosis, e.g., starting lower doses to patients with poorer clinical conditions, did not occur and likely did not influence the median PFS.

## Conclusion

This retrospective multinational cohort study suggests that reduced-dose lenvatinib offers antitumor efficacy in patients with advanced, progressive grade 1–3 GEP and thoracic NET, comparable to the high response rates observed in the TALENT trial with the standard 24 mg/day dose, but with less toxicity. By using lower doses (average of 10–12 mg/day), our findings indicate a more favorable toxicity profile than the significant dose reductions and interruptions reported in TALENT, potentially enhancing patient tolerability and reducing treatment costs. These results highlight the importance of real-world evidence to inform clinical practice.

## Supplementary materials











## Declaration of interest

There is no conflict of interest that could be perceived as prejudicing the impartiality of the research reported.

## Funding

This research did not receive any specific grant from any funding agency in the public, commercial, or not-for-profit sector.

## Ethics

The study was conducted in accordance with the Declaration of Helsinki and local regulations. Institutional review board/ethics committee approval was obtained at each participating center, with a waiver of informed consent owing to the retrospective design and use of de-identified data: AC Camargo Cancer Center, Instituto Alexander Fleming, Hospital Moinhos de Vento, and Hadassah Medical Center.
