# Supplementary material for: Real-world outcomes of lower lenvatinib doses in advanced neuroendocrine tumors: a multinational retrospective study
Source: Endocr Oncol. 2025 Dec 3;5(1):e250076. doi: 10.1530/EO-25-0076 (PMC12679957; doi:10.1530/EO-25-0076)
Supplement: Supplementary file 1 [file supplementary_figure_1.pdf]

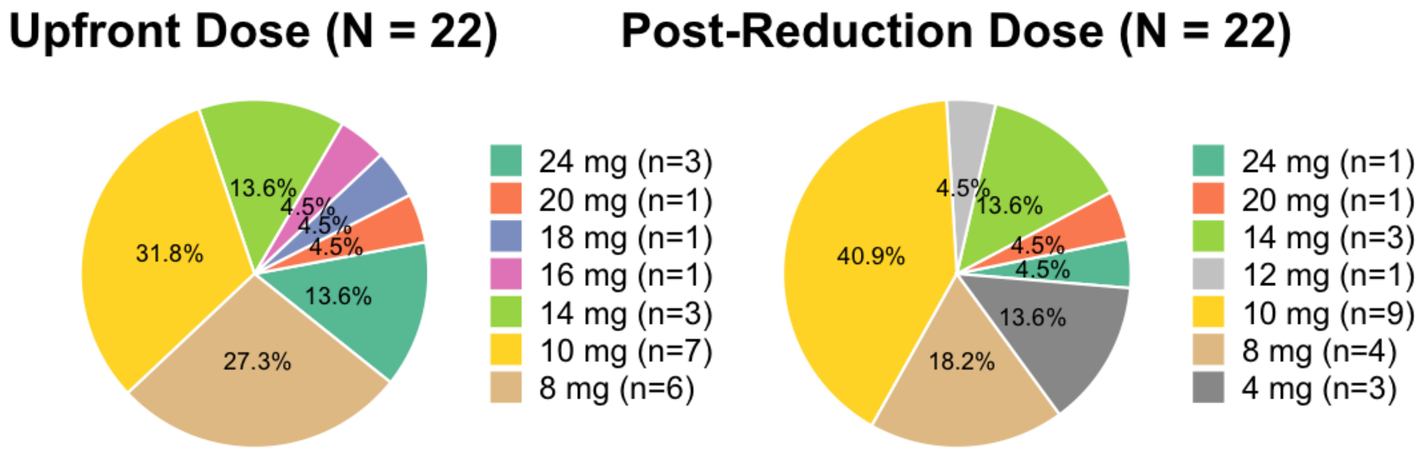

Supplementary figure 1. Distribution of lenvatinib starting and post-reduction doses. The pie charts show the distribution of initial starting doses of lenvatinib (left) and the doses after reduction (right) among 22 patients. The most frequent upfront doses were 10 mg (32%) and 8 mg (27%), whereas after dose adjustment, 10 mg (41%) and 8 mg (18%) predominated.
