# Supplementary material for: Real-world outcomes of lower lenvatinib doses in advanced neuroendocrine tumors: a multinational retrospective study
Source: Endocr Oncol. 2025 Dec 3;5(1):e250076. doi: 10.1530/EO-25-0076 (PMC12679957; doi:10.1530/EO-25-0076)
Supplement: Supplementary file 2 [file supplementary_figure_2.pdf]

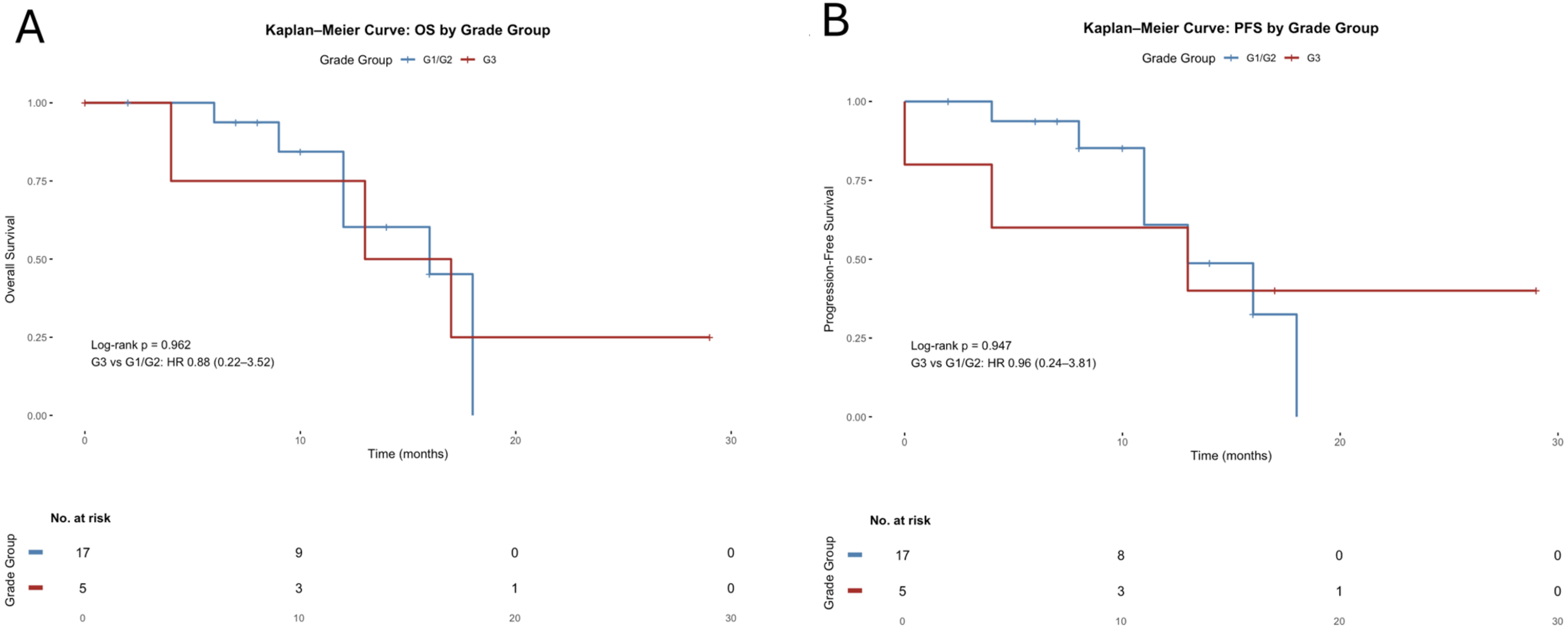

Supplementary figure 2. Survival According to Tumor Grade. Kaplan–Meier curves show overall survival (Panel A) and progression-free survival (Panel B) stratified by tumor grade (G1/G2 vs. G3). No significant differences were observed between grade groups. Numbers at risk at each time point are shown below the plots.
