# Supplementary material for: Real-world outcomes of lower lenvatinib doses in advanced neuroendocrine tumors: a multinational retrospective study
Source: Endocr Oncol. 2025 Dec 3;5(1):e250076. doi: 10.1530/EO-25-0076 (PMC12679957; doi:10.1530/EO-25-0076)
Supplement: Supplementary file 3 [file supplementary_figure_3.pdf]

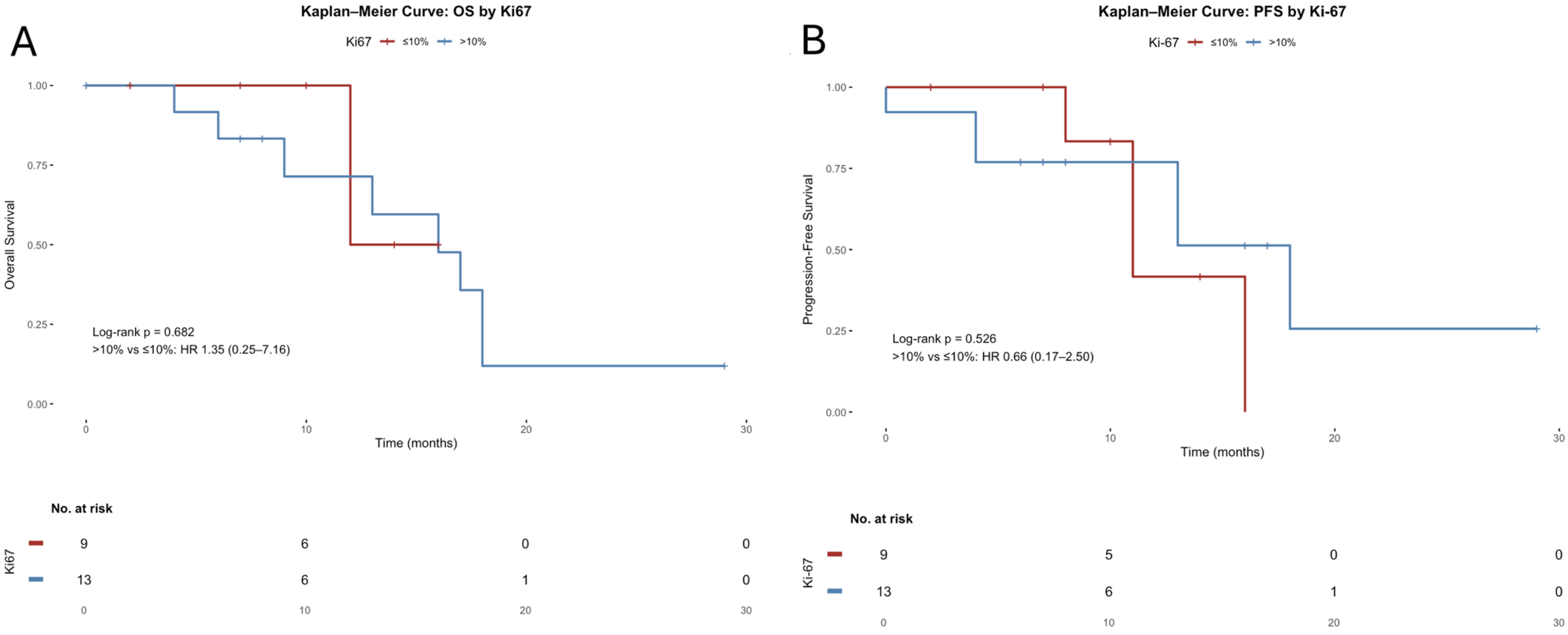

Supplementary figure 3. Survival According to Ki-67 Index. Kaplan–Meier curves show overall survival (Panel A) and progression-free survival (Panel B) stratified by Ki-67 index (≤10% vs. >10%). No statistically significant differences were observed between groups. Numbers at risk are shown below each plot.
