# Supplementary material for: Real-world outcomes of lower lenvatinib doses in advanced neuroendocrine tumors: a multinational retrospective study
Source: Endocr Oncol. 2025 Dec 3;5(1):e250076. doi: 10.1530/EO-25-0076 (PMC12679957; doi:10.1530/EO-25-0076)
Supplement: Supplementary file 4 [file supplementary_figure_4.pdf]

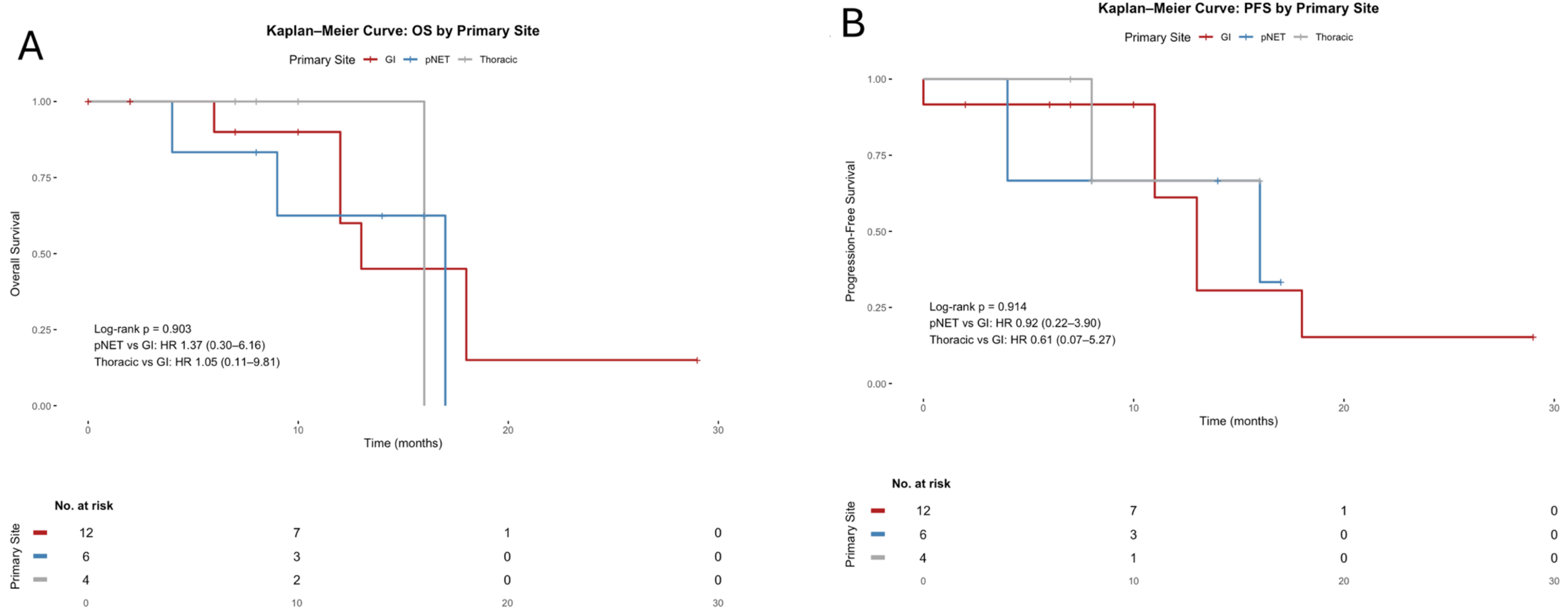

Supplementary figure 4. Survival According to Primary Tumor Site.
Kaplan–Meier curves show overall survival (Panel A) and progression-free survival (Panel B) stratified by primary tumor site (gastrointestinal, pancreatic, or thoracic). No significant differences were observed among groups. Numbers at risk at each time point are shown below the plots.
