# Supplementary material for: Real-world outcomes of lower lenvatinib doses in advanced neuroendocrine tumors: a multinational retrospective study
Source: Endocr Oncol. 2025 Dec 3;5(1):e250076. doi: 10.1530/EO-25-0076 (PMC12679957; doi:10.1530/EO-25-0076)
Supplement: Supplementary file 5 [file supplementary_tables.pdf]

Supplementary table 1. Individual patient characteristics

ID	Primary Site	Grade	Ki-67 (%)	Lenvatinib Line	Upfront Dose (mg)	Dose reduction (mg)	Best Response	Grade 3/4 Toxicity	Adverse Events	Metastatic Sites
1	Small bowel	G2	15	4	10	yes (10→8)	PR	No	G1 Hypertension, G1 Fatigue, G1 Diarrhea	Liver, nodes, bone
2	Small bowel	G2	16	3	24	yes (24→20)	SD	Yes	G3 AST/ALT elevation, G1 Fatigue	Liver
3	Small bowel	G1	1	3	10	no	SD	No	G2 Hepatotoxicity	Liver, bone
4	Small bowel	G3	25	4	8	no	PR	No	G1 Fatigue	Liver
5	Small bowel	G2	7	3	14	yes (14→10)	SD	Yes	G3 Thrombocytopenia, G2 Fatigue	Liver, nodes
6	Small bowel	G2	1	7	24	yes (24→14)	SD	Yes	G3 Vomiting	Liver, nodes
7	Pancreas	G3	40	5	10	no	SD	No	G1 Hypertension	Liver, nodes, bone
8	Pancreas	G2	3	4	24	no	SD	No	NA	Liver
9	Pancreas	G2	10	6	16	yes (16→10)	PR	No	G1 HFS	Liver, nodes
10	Pancreas	G3	25	4	14	no	PD	No	G2 Fatigue, G2 Nausea	Liver, bone
11	Pancreas	G2	15	4	18	yes (18→12)	PR	Yes	G3 Thrombocytopenia, G2 Fatigue	Liver
12	Pancreas	G2	13	5	14	no	SD	No	G2 Fatigue, Myelotoxicity	Liver, nodes
13	Rectum	G2	15	6	20	yes (20→10)	PD	No	G2 Fatigue	Nodes, lung, bones
14	Rectum	G2	15	4	10	no	SD	No	NA	Liver
15	Rectum	G2	10	6	8	yes (8→4)	PR	No	G1 Mucositis	Liver, nodes, bone
16	Rectum	G1	2	4	8	no	SD	No	G1 Fatigue	Liver, nodes, lung, bone
17	Lung (AC)	G2	10	3	10	no	PR	No	NA	Liver, bone
18	LC with high Ki67	G3	30	5	10	no	PD	No	G1 Hypertension, G1 HFS	Nodes, bone
19	Lung (AC)	G2	9	6	8	yes (8→4)	SD	Yes	G4 Myelotoxicity, G1 Fatigue	Liver, nodes, lung, bone
20	ThC with high Ki67	G3	40	3	10	no	PR	No	NA	Nodes, lung, bones
21	Colon	G3	30	5	8	no	SD	No	Hypertension, G1 Fatigue	Liver, nodes, bone
22	Colon	G3	23	6	8	yes (8→8 and 4)	SD	No	NA	Liver

* **Primary tumor site:** LC = Lung Carcinoid; ThC = Thymic Carcinoid; AC = Atypical Carcinoid; **Lenvatinib Line:** Line which Lenvatinib was started.

Supplementary table 2. Number of prior lines of therapy

Number of prior systemic lines	N of patients (%)
2–3 lines	5 (23%)
4–5 lines	11 (50%)
6–7 lines	6 (27%)
**Median (range)**	**4 (3–7)**

S1. Distribution of systemic treatments received before initiation of lenvatinib.
Patients had a median of 4 prior lines (range, 2–7), with most having received 4–5 lines.

Supplementary table 3. Prior treatments

**Therapy Type**	**N (%) of patients (n=22)**
Somatostatin analogue (Octreotide / Lanreotide)	22 (100%)
PRRT (^{177}Lu-DOTATATE)	9 (41%)
Everolimus	9 (41%)
Chemotherapy (CAPTEM, DTIC-5FU, platinum regimens)	14 (64%)
Sunitinib	3 (14%)
Interferon	1 (5%)
Pembrolizumab	1 (5%)
Pazopanib	1 (5%)

S3. Systemic therapies received before lenvatinib initiation.
All patients had been treated with a somatostatin analogue. PRRT, everolimus, and chemotherapy were the most frequent treatments.
